# Structural Behavior of High Durability FRP Helical Screw Piles Installed in Reclaimed Saline Land

**DOI:** 10.3390/polym16121733

**Published:** 2024-06-19

**Authors:** Sun-Hee Kim, Hyung-Joong Joo, Wonchang Choi

**Affiliations:** 1 Department of Architectural Engineering, Gachon University, Sujeong-gu, Seongnam-si 13120, Republic of Korea; shkim6145@gachon.ac.kr; 2 Trinasolar, Jongro-gu, Seoul 03142, Republic of Korea; hyung-joong.joo@trinasolar.com

**Keywords:** fiber-reinforced plastic, helical screw pile, finite element analysis, parametric analysis

## Abstract

The bearing capacity of fiber-reinforced plastic (FRP) helical screw piles is determined by the lesser of the breaking load at the bolted joint and the resistance provided by the screw tip area. In this study, compression and tensile tests were performed with the number of bolts and edge distance as variables. It showed similar strength when compared to the failure stress derived from material testing. In addition, considering load resistance performance, the optimal screw cross section was obtained through parametric analysis. Considering the structural behavior of the screw, a prediction equation was presented to design the screw cross-section as a tapered cross-section using a theoretical method. As a result of comparing the screw cross-section with the finite element analysis results, it was confirmed that the design stress and analysis stress showed an error of 1.1 MPa and were within the allowable stress of 80 MPa.

## 1. Introduction

South Korea announced the “2050 Agri-Food Carbon Neutrality Promotion Strategy” to achieve carbon neutrality in the agricultural sector. The plan aims to reduce fossil fuel usage and increase the supply of renewable energy by transitioning the energy sources for agriculture and rural areas to renewable sources, with the expansion of solar energy prioritized by utilizing agricultural production infrastructure, reclaimed farmland with high soil salinity, and agricultural facilities. Specifically, land reclaimed from the sea, characterized by high clay content, poor drainage, and high salinity, poses challenges for agricultural use. These idle sites should be efficiently utilized by introducing high-value industries while avoiding damage to the land from the destruction of natural ecosystems. This study focuses on the piles used to support photovoltaic (PV) structures on reclaimed land. A PV plant requires a large land area for installation, and piles must be installed to prevent land damage. Traditional painted steel piles are susceptible to damage during ground penetration, which exposes the steel and leads to rapid corrosion, potentially resulting in heavy metals leaching into the soil. Hence, foundations that can replace reinforced concrete and steel are necessary to effectively use idle reclaimed land in the future. The foundation of a structure is crucial for transferring loads to the ground and requires stiffness and strength beyond the specified performance. Therefore, composite materials are considered suitable alternatives to conventional concrete and steel. Owing to the rapid construction requirements of solar power plants, we developed fiber-reinforced plastic (FRP) helical screw piles using a screw pile method that excels in securing construction, drawing, and compression performance through dry construction. The screw pile, originally a type of helical pile with wings attached to a small steel pipe, has been redeveloped using composite materials instead of steel pipes. Extensive research in South Korea has explored the bearing capacity characteristics of conventional helical piles through indoor model experiments and field load tests [1,2,3,4,5,6]. Additionally, Jeong [7] conducted a field applicability evaluation of helical piles with hexagonal joints, finding that the bearing capacity according to the AC 358 code exceeded 600 kN. Bae et al. [8] analyzed the vertical and horizontal behaviors of helical piles in soft ground, focusing on the helix shape and position.

Internationally, Lanyi-Bennett et al. [9] performed compression tests on helical piles in a clay ground environment to study their behaviors. Wang et al. [10] both theoretically and experimentally confirmed the impact on the uplift capacity of single-plate helical piles. Mooney et al. [11] researched the pullout resistance characteristics of single and multiple rotary penetration anchors in sandy and cohesive soils, discussing the relationship between extreme pullout resistance and the installation dimensions of rotary penetration piles. Rao et al. [12] investigated the factors influencing the pullout force of rotary penetration anchors. Merifield [13] numerically analyzed the ultimate pullout resistance of rotary penetration anchors installed in clay and explored the relationship between ultimate pullout resistance and the installation dimensions of rotary penetration piles.

Ductile iron piles, known as screw piles or rotary penetration pipe piles, provide support by penetrating the ground without displacement through rotational force. Previous research on these piles has primarily focused on steel and concrete piles. Mansour et al. [14] also confirmed the shear behavior of recycled aggregate concrete piles reinforced with CFRP through experiments and finite element analysis. FRP is mainly used as a stiffening member, and cases of its use as a main member are extremely rare.

This study developed FRP-applied screw piles in response to soil contamination issues associated with reinforced concrete foundations and steel screw piles used in conventional PV power generation facilities. Owing to the use FRP as a main member, the mechanical properties of FRP should be confirmed. The mechanical properties of FRP were confirmed through compressive and tensile tests. Considering screw piles are driven into the ground by rotation, the torsional performance of the pile body and the adhesion of the helical disk to the body are important considerations. Considering the rapid construction schedule of PV power plants, lightweight pile foundations are mostly used owing to the relative difficulty of applying wet construction methods such as reinforced concrete. Pile foundations manage gravity-directed loads through skin friction and tip bearing capacity, primarily relying on skin friction for pullout resistance, which enhances their performance against pullout loads. This study examined the structural behavior and properties of small-diameter pile foundations used in lightweight structures like PV power plants. Moreover, considering the composite material and the superstructure are bolted together, the compression and tensile resistance performance of the composite material body and the connection are key structural variables in screw pile design. Therefore, the structural safety of the pile was confirmed through load resistance tests and finite element analysis.

## 2. FRP Helical Screw Pile

### 2.1. FRP Helical Screw Pile Shape

The screw pile used in PV power plants is constructed by fabricating a separate screw for a round steel pipe and then joining the steel pipe and screw via welding. The dimensions of the steel pipes used as pile bodies vary based on the soil conditions. Typically, these piles have diameters ranging from 70 to 100 mm, thicknesses from 3.5 to 7.5 mm, and lengths from 600 to 4000 mm, depending on the required load resistance performance. Moreover, the diameter of the screw blade is generally set between approximately 2.0 and 5.0 times the diameter of the shaft, with the earth load on the blade increasing as the diameter increases.

The dimensions of screw piles are often based on the empirical experience of each manufacturer rather than on precise theoretical knowledge or assessments. Once the installation site for the power plant is determined, the load resistance performance is evaluated through field tests and subsequently applied to the design. In this study, a high-durability screw pile, designed for use in salty and wet ground environments, was conceptualized in shapes similar to those of conventional screw piles. To establish the basic dimensions for FRP helical screw piles (HANKUK FIBER, Miryang-si, Republic of Korea), their mechanical properties and torsional stiffness were compared with those of conventional steel screw piles, as detailed in Table 1 and Table 2.

The shear modulus values in Table 1 were determined considering the characteristics of each material by the following equation:

(1) $$G_{xy}=\frac{E_{x}E_{y}}{E_{x}{+E}_{y}+2E_{y}\nu_{xy}},$$

where the subscripts x and y denote the directions; these values are equal for isotropic materials such as steel. Furthermore, E and ν represent the elastic modulus and Poisson’s ratio, respectively. The mechanical properties and dimensions of conventional steel screw piles were utilized to determine the cross-sectional dimensions of the pile, as outlined in Table 1. Considering the screw piles are installed through rotary penetration, they are designed to exhibit torsional stiffness comparable to that of conventional steel pipe screw piles. The relationship between the applied torque and the torsional angle is a function of the torsional stiffness GJ, which can be expressed mathematically as follows:

(2) $$\phi =\frac{TL}{GJ\;},$$

where *T* and *L* denote the torque and length of the pipe, respectively; *G* denotes the shear modulus; and *J* denotes the torsional constant. The torsional constant can be calculated for a circular tube as follows:

(3) $$J=2\pi r^{3}t,$$

As indicated in Table 2, torsional stiffness significantly influences the cross-sectional dimensions. Composite materials require larger cross-sectional dimensions because they possess substantially lower circumferential shear moduli compared to steel. Based on the findings in Table 2, the basic cross-sectional dimensions for the pilot prototype of the FRP helical screw pile body were 150 mm in diameter, with a thickness ranging from 6.0 to 8.0 mm.

### 2.2. Configuration of FRP Helical Screw Piles

**Figure 1:**
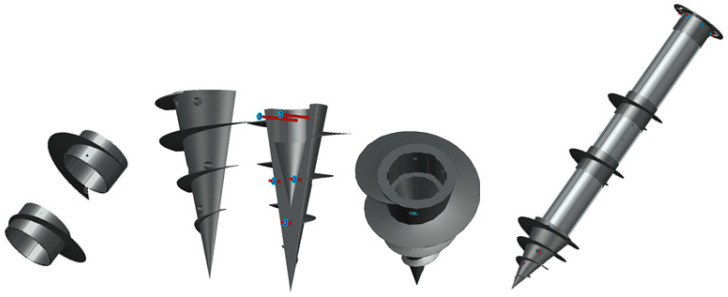
FRP helical screw piles.

FRP helical screw piles are composite round tubes equipped with screws made of stainless steel, steel molding compounds (SMC), or FRP. Figure 1 illustrates the various shapes of FRP helical screw piles.

The tip screws are vertically separated and attached to the round tube using stainless steel (STS) bolts. The screws in the center are oriented horizontally and fastened to the round tube with STS bolts.

### 2.3. Failure Mode for Composite Connections

When designing a pile, considering the connections and the structural safety of these connections is crucial. The strength of a bolted connection is influenced by several factors. For instance, in the construction field, the clearance around bolt holes must be sufficiently large to facilitate construction. However, no design standards for composite connections have been established in South Korea. Therefore, international standards were reviewed, with the bolt-hole clearance of 1.6 mm (as proposed by the American Society of Civil Engineers (ASCE) [15]) adopted for the developed FRP helical screw piles to ensure it surpasses the values suggested by the design standards.

**Figure 2:**
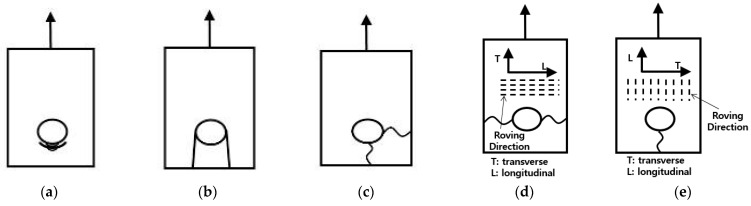
Failure modes of bolts: (**a**) bearing failure; (**b**) shear-out failure; (**c**) block-shear failure; (**d**) net-tension failure; (**e**) cleavage failure.

The failure modes of bolted composite connections are classified into bearing failure, shear-out failure, block-shear failure, net-tension failure, and cleavage failure, as depicted in Figure 2 [16,17,18,19]. Figure 2a shows a bearing failure, which occurs as a gradual fracture of the cross-section of the member’s bolt hole where it contacts the neck of the bolt under load, leading to a reduction in cross-sectional area. Figure 2b displays a shear-out failure, where the area around the bolt neck advances further into bearing failure before failing along the shear plane due to reduced shear resistance. Block-shear failure, illustrated in Figure 2c, represents the simultaneous occurrence of shear failure and net-tension failure, resulting in a part of the member tearing off. Figure 2d shows a net-tension failure, where the reduction in cross-sectional area caused by the bolt holes leads to the tensile stress from the load surpassing the tensile strength of the member material, causing failure along the line of bolt arrangement. Finally, Figure 2e depicts a cleavage failure, where the bolt hole section of a member that receives a load from the neck of the bolt fails to withstand the load and fractures.

## 3. Structural Performance Evaluation of FRP Helical Screw Piles

### 3.1. Compressive Strength Test and Results

**Figure 3:**
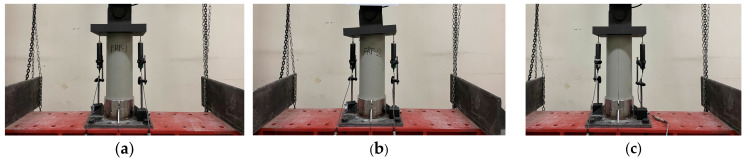
Compressive strength test of FRP body: (**a**) FRP_S_1; (**b**) FRP_S_2; (**c**) FRP_S_3.

The compressive strength test was conducted on a cylindrical tube intended as the main body of the FRP helical screw pile. The compressive strength test was performed according to KS F 2405 [20]. To examine the compressive strength of the FRP cylindrical tube, the experimental variables of the FRP compressive strength test are shown in Table 3. This tube was 150 mm in diameter, 500 mm in length, and 4.5 mm in thickness, as detailed in Table 3. Three specimens were prepared to evaluate its performance under compression. During the test, a displacement meter with a 50-mm capacity was positioned at the center of each specimen to measure longitudinal compressive displacement, as illustrated in Figure 3. A steel plate was placed atop each specimen to evenly distribute the load. The loading was performed at a rate of 3 mm per minute using the displacement control method on a universal testing machine (UTM).

**Figure 4:**
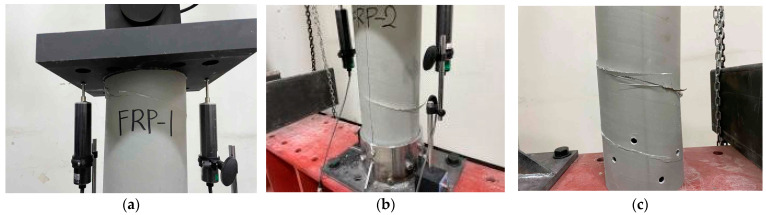
Compressive strength test results of the FRP body: (**a**) FRP_S_1; (**b**) FRP_S_2; (**c**) FRP_S_3.

**Figure 5:**
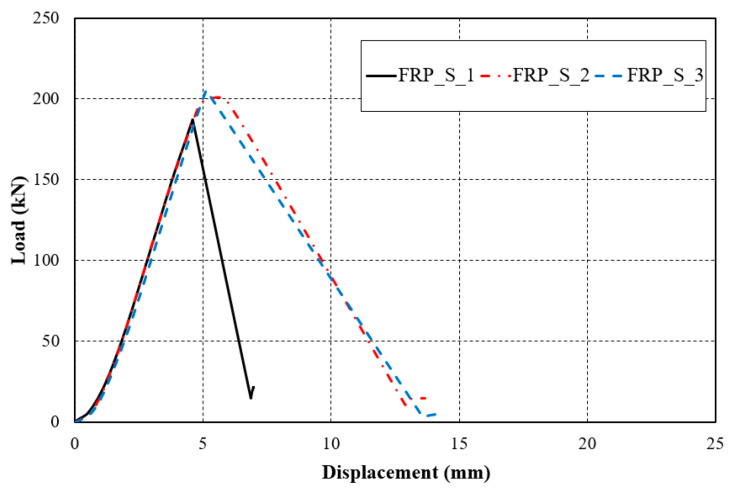
Load–displacement relation for FRP cylindrical tubes.

In the compression resistance performance test of the FRP helical screw pile body, all three test specimens failed due to cracking in the circumferential direction, aligned with the placement of the reinforcing fibers, as illustrated in Figure 4. Upon reapplication of the compressive load, the load–displacement relationship remained linear until material failure, as indicated by the displacement meter shown in Figure 5. The load resisted up to 186.87 kN, 198.03 kN, and 205.40 kN, respectively, and the load decreased as the specimen fractured. The failure load for all specimens was consistently around 186 kN, as documented in Table 4. This strength was comparable to the failure stresses determined through material testing.

### 3.2. Tensile Test and Results

**Figure 6:**
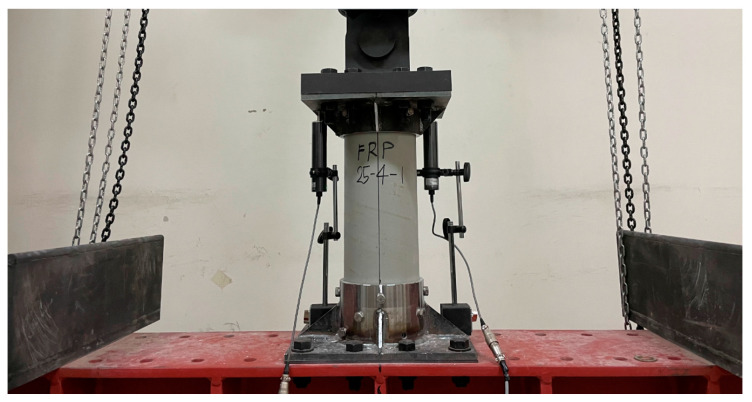
Tensile test of the FRP.

**Figure 7:**
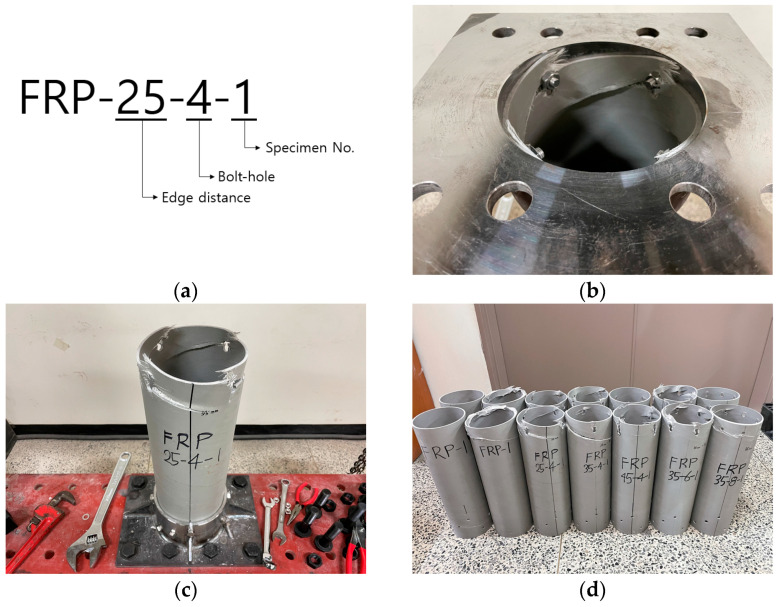
Failure modes of tensile test specimens: (**a**) detail of specimen; (**b**) failure mode within upper jig; (**c**) failure mode of FRP-25-4-1; (**d**) failure mode of tensile test all specimens.

The composite material and the superstructure are connected by bolts. The tensile resistance performance of the composite body and joints is the most important structural variable in the design of FRP screw piles. This load resistance performance was evaluated through the tensile strength test. To assess the tensile strength of the bolted FRP cylindrical tube, a tensile test was conducted with variations in edge distance and the number of bolts. Specific jigs were constructed for securing the top and bottom of each specimen using M10 STS bolts through the pile body, complemented by stainless steel bolts, flat washers, and nuts of the same material. The specifications of the tested specimens are detailed in Table 5, with two samples tested for each configuration. Each specimen was loaded in 100 tonf-capacity UTM with a displacement meter positioned at the center, as depicted in Figure 6, applying the load at a rate of 3 mm/min using the displacement control method. The modulus of elasticity for FRP was determined from the strain slope in the 1000–3000-μm range following the procedure outlined by ASTM D3039/D3039M [21]. The failure morphologies resulting from the tensile tests are shown in Figure 7a–c.

In the tensile tests, all specimens exhibited separation from the composite at the bolted area, aligned with the direction of the reinforcing fibers, and ultimately failed at the bolted connection. Figure 7a–c displays the failure morphologies of the tensile test specimens. The fracture strength of the connections, influenced by variables such as the number of bolts and edge distance, is summarized in Table 6. According to the material test results, the failure load of the FRP pile body is approximately 164.6 kN. However, the strength of the connection was evaluated to be a maximum of 96.96 kN when 8 bolts were used, and it was confirmed that failure occurred in the bolt connection.

### 3.3. Numerical Analysis of the FRP Helical Screw Pile

**Figure 8:**
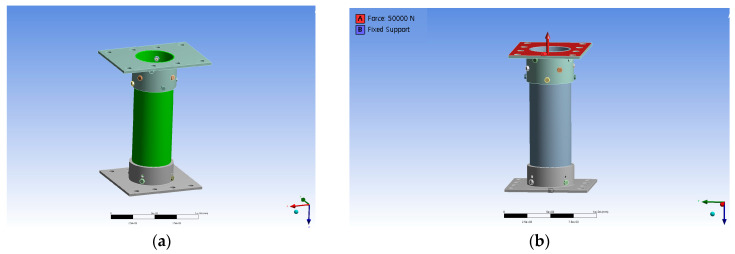
Finite element analysis: (**a**) modeling; (**b**) boundary conditions and load conditions.

The finite element analysis of the bolted connection in the FRP helical screw pile was conducted using ANSYS Workbench Ver. 19.2 [22], a general-purpose finite element analysis software. The finite element model, matching the experimental specimen dimensions of 150 mm outer diameter, 500 mm length, and 4.5 mm thickness, is shown in Figure 8a. The mechanical properties of specimens used in the finite element analysis are given in Table 1. The model was fixed at the base, and a vertical load was applied from the top as depicted in Figure 8b, gradually increasing to evaluate the stress at the bolted connections and compare it with experimental results. The model comprised 437,966 nodes and 130,337 elements, with an element size of 250 mm. The element size of the FRP helical screw pile was assumed to be 15 mm.

**Figure 9:**
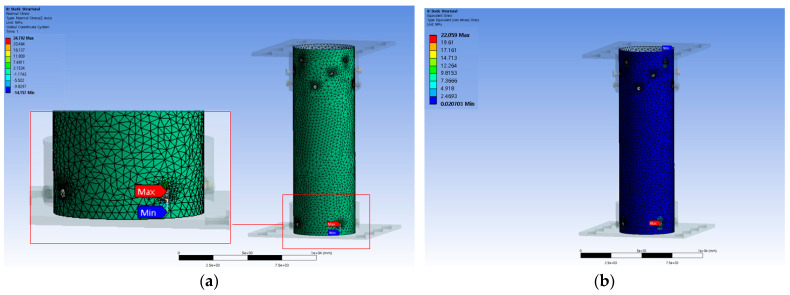
Example of finite element analysis results (specimen FRP-25-4): (**a**) stress distribution; (**b**) von Mises stress.

Finite element analysis indicated that maximum stresses occurred around the bolt, as demonstrated in Figure 9a, which shows the stress distribution. As shown in Figure 7c, this is the same as the fracture occurring around the bolt hole through the tensile strength test. The failure stress of specimen FRP-25-4 was 20.70 MPa, and the finite element analysis results showed that the breaking stress of FRP-25-4 was 22.059 MPa. Figure 9b illustrates the von Mises stress within the FRP, determined through finite element analysis. An error of approximately 6.16% was noted between the failure stress obtained experimentally and that estimated via the finite element analysis, indicating that the failure was due to stress concentration around the bolt. Furthermore, the finite element analysis provided an estimate of the failure stress of the FRP.

### 3.4. Analysis of Experimental Results of Bolted Connections in FRP Helical Screw Piles

**Figure 10:**
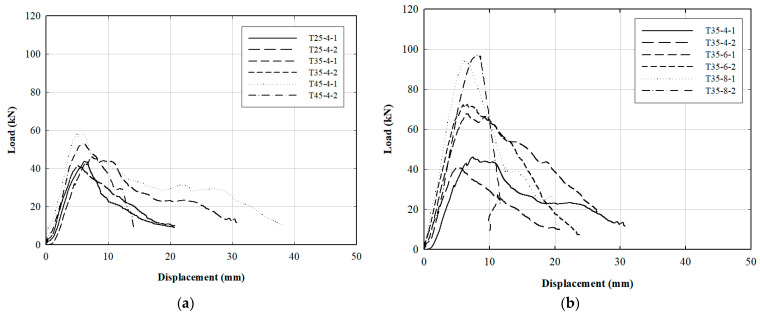
Experimental results for bolted connection of FRP helical screw pile: (**a**) load–displacement relation for FRP by edge distance; (**b**) load–displacement relation for edge distance of 35 mm.

The results of the bolted connection test for the FRP helical screw pile, with edge distance as a variable, are displayed in Figure 10. Figure 10a shows that an edge distance of 45 mm provided a 30.8% higher resistance to load compared to when bolts were 25 mm apart, and a 16.3% increase in strength over a 35 mm edge distance. Furthermore, the tensile behavior of the FRP pile was analyzed by varying the number of bolts at a 35 mm edge distance. As depicted in Figure 10b, a load resistance of 95.80 kN was observed with eight bolts fastened, which ultimately led to net-tension failure characterized by tearing at the bolt holes. However, with six bolts at a 35 mm edge distance, the FRP pile exhibited a shear-out failure where the FRP shear plane failed, progressing from the bearing failure. The failure loads and modes for the FRP bolted connections are summarized in Table 6. Shear-out failure was predominant; the member adjacent to the bolt neck developed bearing failure and subsequently failed along the shear plane. Despite varying edge distances, the overall pattern of the failure mode showed that narrow member widths did not significantly affect the failure mode or strength; most shear failures occurred regardless of edge distance, but fracture strength increased as edge distance increased. The load of FRP for each edge distance in Figure 10b showed a tendency to increase. Similar to the results of previous studies [18,23,24], the fracture strength tended to increase as the edge distance of the bolt hole increased.

**Figure 11:**
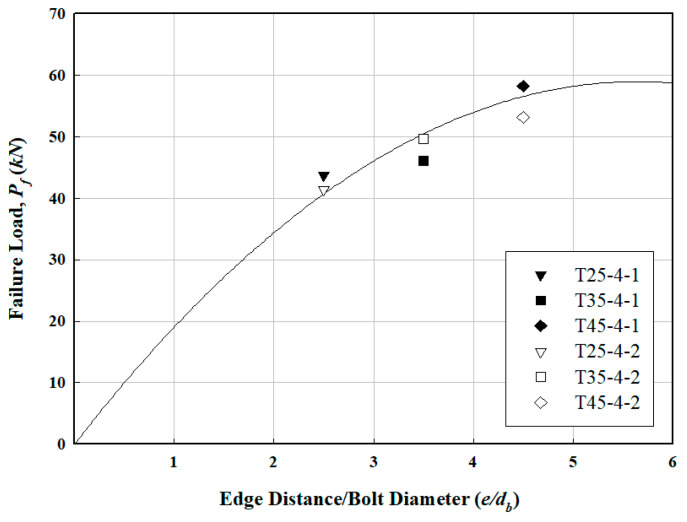
Relation between failure load and member edge distance of FRP bolted connection specimens.

**Figure 12:**
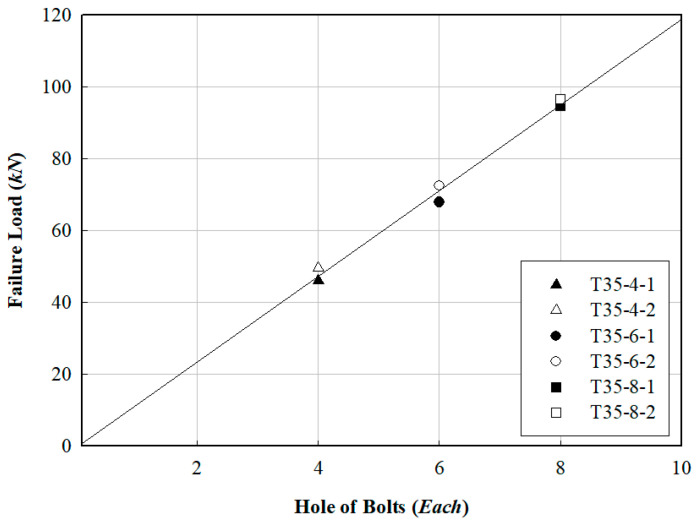
Relation between failure load and number of bolts in FRP specimens.

The FRP specimen with four bolts showed a consistent increase in failure load as the edge-to-bolt diameter ratio ($e/d_{b}$) increased to 2.5, 3.5, and 4.5. Figure 11 illustrates the relationship between failure load and member edge distance, while Figure 12 depicts the load variation as a function of the number of bolts at an $e/d_{b}$ of 3.5. Although the load increased with the number of bolts from four to eight, the FRP specimen at an $e/d_{b}$ of 3.5 experienced tensile failure with eight bolts, indicating that excessive bolt tightening did not prevent tensile failure. According to a previous study by Lee et al. [17], shear failure occurred in most cases as $e/d_{b}$ increased. According to previous research [17,23], an appropriate specimen width should be applied to prevent tensile failure in the cross-section. We therefore recommended that the FRP helical screw pile developed herein use between 4 and 6 bolts.

## 4. Performance Evaluation of FRP Helical Screw Piles

### 4.1. Body and Screw Designs

**Figure 13:**
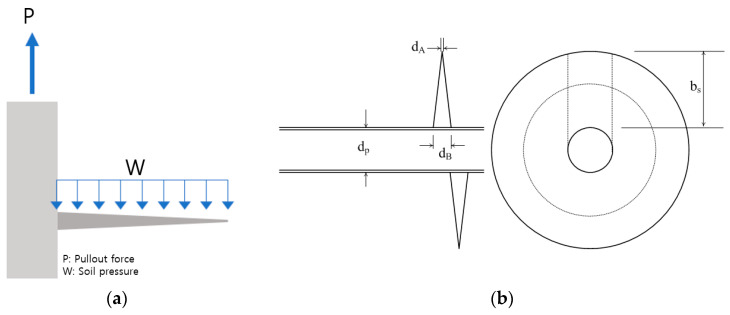
FRP screw pile: (**a**) load transfer structure; (**b**) design of FRP screw piles.

The proposed site for the saltwater PV power plant consists of landfill and sedimentary layers—characterized by loose and ineffective ground. Therefore, the bearing capacity of the screw pile is primarily determined by the pile geometry rather than the interaction between the ground and pile [25]. Considering the screw in an FRP helical screw pile transfers the load from the pile to the soil, the entire tip resistance area of the screw is assumed to resist the soil. The screw wing can be considered as a cantilever fixed to the body if the inclination of the screw to the thread is negligible. Moreover, when a uniform load is applied to a cantilever, the free end requires less load resistance than the fixed end, making it effective to apply the wing of the screw to the end face. A uniformly distributed load is applied to the screw as shown in Figure 13a. The design of the FRP screw pile is as shown in Figure 13b.

The stress on the cross-section is a function of the moment of inertia. The moment of inertia for a variable cross-section can be expressed as follows [26]:

(4) $$I_{x}={I_{A}\left[1+\left(\frac{d_{B}}{d_{A}}-1\right)\frac{x}{L_{s}}\right]}^{n},$$

where *d_A_* and *I_A_* are the height of the cross-section at the free end and the secondary moment of the cross-section, respectively, and *d_B_* is the height at the fixed end. Further, *n* is the shape factor and is defined as follows:

(5) n=log⁡IBIAlog⁡dBdA ,

The bending stress acting on the screw wing can be expressed as follows:

(6) $$\sigma_{s}=\frac{Px^{4}}{2\pi I_{x}\;}\frac{d_{A}}{d_{p}}\left[1+\left(\frac{d_{B}}{d_{A}}-1\right)\frac{x}{L}\right],$$

where *d_p_* denotes the diameter of the pile body, *d_A_* and *d_B_* denote the cross-sectional height at the screw, and *P* denotes the required pullout force of the FRP helical screw pile. Moreover, *x* and *I_x_* are the moment of inertia at a random point from the tip of the screw wing toward the pile body and that at a random point shown in Equation (4), respectively.

The FRP helical screw piles connect the screw and head reinforcement using bolts. Therefore, the strength of this connection is a crucial design variable that influences the structural performance of the pile. Considering the load resistance performance and uniformly distributed load applied to the screw according to the diameter of the body, the screw dimensions were presented so that the stress generated in the screw does not exceed the allowable stress. For the bending stress in Table 7, Equation (6) was applied using the second moment of inertia shown in Equation (4). The minimum number of bolts for screw and head stiffeners is recommended based on the lowest stresses observed in the connection from the structural tests of bolted connections presented in Section 3. The dimensions of the FRP helical screw pile and bolt quantities are detailed in Table 7.

### 4.2. Screw Cross-Section Analysis

**Figure 14:**
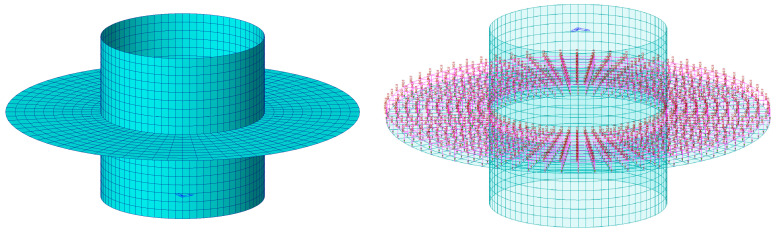
FRP screw analysis modeling.

A structural analysis was conducted using the general-purpose analysis program MIDAS to assess the design adequacy of the FRP helical screw pile cross-section [27]. Given that the FRP helical screw pile comprises thin plate members, it was modeled using the plate element, as shown in Figure 14, to perform the finite element analysis. Table 1 was used for the mechanical properties of the materials. The mesh was uniformly divided as shown in Figure 14. The mesh of the screw model contained 2161 nodes and 2088 elements. The design requirement of 2 tonf includes the soil load applied to the screw when embedding screw piles. Therefore, the behavior of the screw was confirmed without modeling the soil in the finite element analysis. The analysis focused on a cross-section of the screw required for a pullout performance of 2 tonf, confirming the safety of the design.

**Figure 15:**
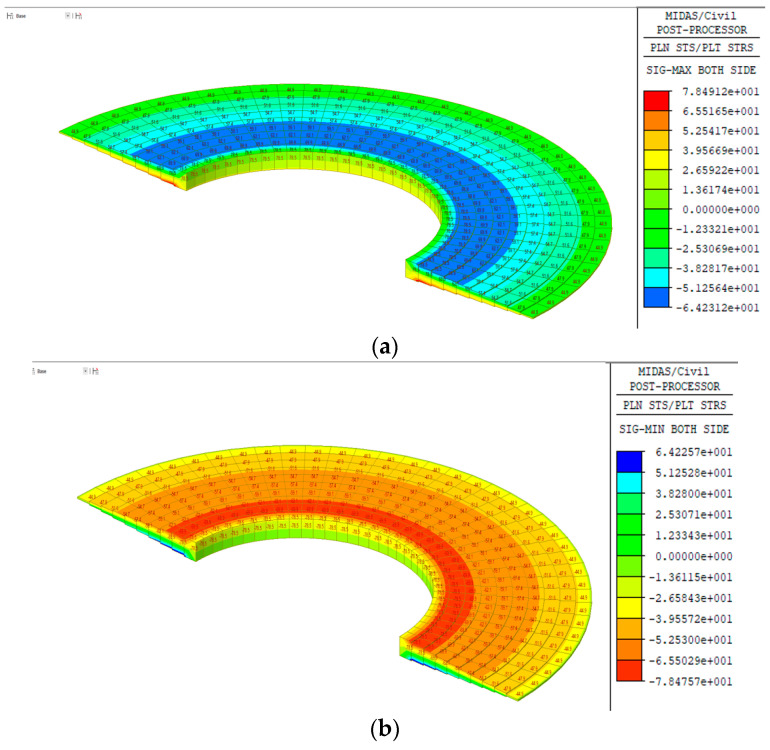
Finite element analysis results of FRP helical screw pile: (**a**) maximum stress; (**b**) minimum stress.

The results of the finite element analysis are shown in Figure 15 and Table 8, revealing that the stresses in the screw were minor compared to the allowable stresses.

## 5. Conclusions

This study outlined the basic design of an FRP helical screw pile, considering the features of traditional helical screw piles—specifically the spiral disc integrated with the main body that rotates and penetrates into the ground—as well as the specifications of commercially available steel helical screw piles. A structural analysis was conducted using the general-purpose analysis program MIDAS to assess the structural integrity of the FRP helical screw pile cross-section.

The compressive strength test results for the FRP helical screw piles indicated a consistent failure load across all test specimens, approximately 186 kN. Tensile testing of the FRP helical screw pile revealed failures predominantly at the bolted connections. The results showed that increasing the section length between the bolt hole and the end of the member tended to shift failure modes from block-shear failure, where part of the member is torn off, to bearing failure. In the tensile tests of FRP helical screw piles with four bolts, shear failure became prevalent as the edge-to-bolt diameter ratio (e/d_b_) increased. At an e/d_b_ of 3.5, shear failure was common in piles with four, six, and eight bolts, but net-tension failure occurred with eight bolts. Consequently, using four bolts is recommended for FRP helical screw piles to mitigate net-tension failure. The influence of FRP helical screw piles on the strength of bolted connections was evaluated through both compression and pullout tests. Additionally, the bearing capacity of FRP screw piles is determined by the smaller of the breaking load of the bolt joint and the resistance due to the screw tip area. Therefore, the optimal cross-section for the FRP screw pile was determined through design considerations and numerical analysis of the FRP helical screw pile structure. In future studies, the FRP helical screw pile will be applied in field settings to verify its practicality and constructability.

## Figures and Tables

**Table 1:** Mechanical characteristics.

Category		Modulus of Elasticity (GPa)	Strength (MPa)	Poisson’s Ratio (mm/mm)	Shear Modulus (GPa)	Remarks
Steel pipe		205	270	0.300	76.9	
GFRP pipe	Hoop	16.5	180	0.159	4.3	Circumferential
	Axial	6.5	80	0.159	3.7	Longitudinal

**Table 2:** Dimensions of the helical screw pile body and torsional stiffness.

Category	Diameter (mm)	Thickness (mm)	*GJ* (kN·m^2^)	Remarks
Steel pipe	76.0	3.0	79.5	Commercial product
		3.5	92.8	
		4.5	119.3	
GFRP pipe	150.0	3.0	34.2	
		4.0	45.6	
		5.0	57.0	
		6.0	68.4	
		7.0	79.8	
		8.0	91.2	
	125.0	3.0	19.8	
		4.0	26.4	
		5.0	33.0	
		6.0	40.0	
		7.0	46.2	
		8.0	52.8	
	100.0	3.0	10.1	
		4.0	13.5	
		5.0	16.9	
		6.0	20.3	
		7.0	23.6	
		8.0	27.0	

**Table 3:** Dimensions of compressive strength test specimens.

Specimen Number	Outer Diameter (mm)	Length (mm)	Body Thickness (mm)	Cross-Sectional Area (mm^2^)
FRP_S_1	150	500	4.35	4350
FRP_S_2	150	500	4.36	4360
FRP_S_3	150	500	4.32	4320

**Table 4:** Compressive strength test results.

Category	Failure Load (kN)	Failure Stress (MPa)		Displacement (mm)
		Experimental Results	Material Experiment	
FRP_S_1	186.87	90.80	80.00	4.60
FRP_S_2	198.03	96.30		5.97
FRP_S_3	205.40	99.60		5.12
Average	196.77	95.57		5.23

**Table 5:** Specifications of the tensile test specimens.

Specimen Number	Outer Diameter (mm)	Length (mm)	Body Thickness (mm)	Bolt Quantity	Edge Distance (mm)
FRP-25-4-1	150	500	4.38	4	25
FRP-25-4-2	150	500	4.35	4	25
FRP-35-4-1	150	500	4.36	4	35
FRP-35-4-2	150	500	4.34	4	35
FRP-35-6-1	150	500	4.32	6	35
FRP-35-6-2	150	500	4.40	6	35
FRP-35-8-1	150	500	4.36	8	35
FRP-35-8-2	150	500	4.32	8	35
FRP-45-4-1	150	500	4.35	4	45
FRP-45-4-2	150	500	4.36	4	45

**Table 6:** Tensile test results.

Category	Edge Distance Ratio (e/d_b_)	Failure Load (kN)	Average Load (kN)	Failure Mode
FRP-25-4-1	2.5	43.75	42.58 ± 1.18	block-shear
FRP-25-4-2	2.5	41.40		block-shear
FRP-35-4-1	3.5	46.12	47.90 ± 1.78	shear-out
FRP-35-4-2	3.5	49.67		shear-out
FRP-35-6-1	3.5	67.94	70.25 ± 2.32	shear-out
FRP-35-6-2	3.5	72.57		shear-out
FRP-35-8-1	3.5	94.64	95.80 ± 1.16	net-tension
FRP-35-8-2	3.5	96.96		net-tension
FRP-45-4-1	4.5	58.26	55.73 ± 2.54	shear-out
FRP-45-4-2	4.5	53.19		shear-out

**Table 7:** FRP helical screw pile design results.

No.	Required Performance kN (tonf)	Body Diameter (*d_p_*, mm)	Dimensions			Bending Stress $(\sigma_{s},$ MPa)	Connections (8-mm Bolt Set)		
			Wing Width (*b_s_*, mm)	*d_A_* (mm)	*d_B_* (mm)		Body Thickness (mm)	Bolt Quantity (ea)	Tensile Strength (kN)
1	9.81 (1.0)	100	116	1	9.3	79.0	3.0	4	22.08
							3.5	4	25.76
							4.0	4	29.44
							4.5	4	33.12
2	19.6 (2.0)	100	116	1	13.1	79.6	3.0	4	22.08
							3.5	4	25.76
							4.0	4	29.44
							4.5	4	33.12
3	29.4 (3.0)	125	108	1	14.6	80.0	3.0	5	33.12
							3.5	5	32.20
							4.0	4	29.44
							4.5	4	33.12
4	39.2 (4.0)	150	100	1	15.5	80.0	3.0	8	44.16
							3.5	7	45.08
							4.0	6	44.16
							4.5	5	41.4

**Table 8:** Comparison of FRP screw analysis results.

Category	Design Stress	Analyzed Stress	Δ	Allowable Stress	Review
Bending stress	79.6 MPa	78.5 MPa	1.1 MPa	80.0	O.K.

## Data Availability

The original contributions presented in the study are included in the article, further inquiries can be directed to the corresponding author/s.
